# Extension of biomass estimates to pre-assessment periods using density dependent surplus production approach

**DOI:** 10.1371/journal.pone.0186830

**Published:** 2017-11-13

**Authors:** Jan Horbowy, Maciej T. Tomczak

**Affiliations:** 1 National Marine Fisheries Research Institute, Gdynia, Poland; 2 Baltic Sea Centre, Stockholm University, Stockholm, Sweden; Chinese Academy of Forestry, CHINA

## Abstract

Biomass reconstructions to pre-assessment periods for commercially important and exploitable fish species are important tools for understanding long-term processes and fluctuation on stock and ecosystem level. For some stocks only fisheries statistics and fishery dependent data are available, for periods before surveys were conducted. The methods for the backward extension of the analytical assessment of biomass for years for which only total catch volumes are available were developed and tested in this paper. Two of the approaches developed apply the concept of the surplus production rate (SPR), which is shown to be stock density dependent if stock dynamics is governed by classical stock-production models. The other approach used a modified form of the Schaefer production model that allows for backward biomass estimation. The performance of the methods was tested on the Arctic cod and North Sea herring stocks, for which analytical biomass estimates extend back to the late 1940s. Next, the methods were applied to extend biomass estimates of the North-east Atlantic mackerel from the 1970s (analytical biomass estimates available) to the 1950s, for which only total catch volumes were available. For comparison with other methods which employs a constant SPR estimated as an average of the observed values, was also applied. The analyses showed that the performance of the methods is stock and data specific; the methods that work well for one stock may fail for the others. The constant SPR method is not recommended in those cases when the SPR is relatively high and the catch volumes in the reconstructed period are low.

## Introduction

The International Council for the Exploration of the Sea (ICES) considers only the period for which reliable catch-at-age data are available for the analytical assessment of stock dynamics. This period depends on the stock and, in most cases, refers to the last three to four decades (i.e., from the 1970s onwards), and for only a few stocks (e.g., Arctic cod), it extends to the end of the 1940s. However, knowledge of stock dynamics over longer time spans (e.g., from the 1950s or even from earlier years onwards) could be very useful towards quantifying the effects of the environmental and climate changes that occurred in the 20^th^ century on living resources. Such knowledge could help us to better understand the functioning of the ecosystems and the human impact on it, which in turn would lead to improved management of fisheries e.g. [1–3]. The effects of climate and environmental variability on the catch volume, recruitment and stock dynamics are presented and discussed in many papers, and the time span for such analyses extends as far back as the available data allow [4–7]. Such analyses could benefit from the backward extension of the available stock size estimates, even if the precision of such reconstruction would not be high.

Quantified stock dynamics covering time spans longer than three to four decades could also be helpful for setting precautionary, limit or target reference points and could enable the derivation of good environmental status (GES) indices. Such reference points and indices are required by the international agreements and declarations established in the last decades. The Rio Declaration [8] indicates the need for stock management to be consistent with precautionary approach, while the World Summit on Sustainable Development and the European Commission mandated that exploitation needs to be consistent with the maximum sustainable yield (MSY) [9, 10]. Similarly, the Marine Strategy Framework Directive (MFSD) requires the derivation of indices of good environmental status (GES) for the European marine ecosystems and acts to achieve GES within the next several years [11].

Eero and MacKenzie [12] presented a simple method for biomass reconstruction in the pre-assessment period using catch volume only in that period and analytical assessment results from later years. They used the concept of the surplus production rate (SPR). The SPR is calculated using catch volumes and biomass estimated by analytical stock assessment and is used as an average in backward biomass recalculations. However, the analysis provided by Eero and MacKenzie [12] indicates that for some stocks, the SPR showed significant variation over time. In fact, the SPR may be dependent on stock biomass because at high biomass of a stock, the surplus production is usually low, while it is much higher when biomass of stock is low.

Thus, the goal of the present paper is to develop methods for the reconstruction of stock biomass during the pre-assessment period in cases when the assumption of a constant SPR does not hold. The developed methods will use the results of the analytical stock assessments and catch volumes for years not covered by the assessment. The developed methods have been tested first on stocks with known dynamics and then applied for mackerel in the North-east Atlantic.

## Material and methods

The surplus production rate in year *y* is defined as:

$$SPR_{y}=\frac{B_{y+1}-B_{y}+C_{y}}{B_{y}},$$ (1)

where B is biomass and C is catch volume. Eero and MacKenzie [12] reformulated this equation as

$$B_{y}=\frac{B_{y+1}+C_{y}}{1+SPR},$$ (2)

replacing the time dependent SPR_y_ by its average, SPR. Next, they applied Eq (2) to reconstruct stock biomass for years not covered by the analytical stock assessment using only total catch data and the SPR, estimated from the available assessment. This method will be further referred to as the constant SPR method. Eero and MacKenzie [12] concluded that in specific cases, the SPR may be only slightly dependent on time, so it may be assumed to be constant in Eq (2), and the authors provided some justification for such a thesis. However, in general, the surplus production rate is stock density dependent, as may be seen from the theory of stock-production models. For example, using the discrete version of the Schaefer model [13], the change of biomass within year *y* is:

$$B_{y+1}-B_{y}\;=\;hB_{y}(B_{\infty }-B_{y})-qE_{y}B_{y},$$ (3)

where E is the fishing effort, B_∞_ is asymptotic biomass, q is catchability, and h is parameter expressed as exp(r)/ B_∞_ where r is intrinsic rate of increase. Thus, the surplus production rate is:

$${SPR}_{y}\;=\;\frac{B_{y+1}-B_{y}+qE_{y}B_{y}}{B_{y}}\;=\;h(B_{\infty }-B_{y}),$$ (4)

so the rate is linearly decreasing with biomass. Similarly, for the Fox [14] and Pella and Tomlinson [15] models, the SPR can be presented, respectively, as

$${SPR}_{y}\;=\;h({lnB}_{\infty }-{lnB}_{y})$$ (5)

and

$${SPR}_{y}\;=\;h(B_{\infty }^{n-1}-B_{y}^{n-1}),$$ (6)

showing other possible forms of SPR dependence on stock biomass if the stock dynamics can be approximated by discrete form of classical stock-production models.

### Extension of the Eero and MacKenzie approach

The relationship between the “observed” SPR (i.e., based on biomass estimates from analytical stock assessment) and biomass may be tested, and if such a relationship exists, it may be used to estimate biomass in the pre-assessment era. Thus, in addition to the Eero and MacKenzie [12] method, two other approaches were considered in which, following Eqs (4) and (5), the SPR was dependent on biomass linearly or logarithmically (referred to as linear SPR or logarithmic SPR, respectively):

$${SPR}_{y}\;=\;aB_{y}+b$$ (7)

and

$${SPR}_{y}\;=\;alnB_{y}+b,$$ (8)

where a and b are parameters to be determined from the relationship between the observed SPR and biomasses. Coupling Eqs (1) and (7) or (8), we obtain

$$\frac{B_{y+1}+C_{y}-B_{y}}{B_{y}}\;=\;aB_{y}+b$$ (9)

and

$$\frac{B_{y+1}+C_{y}-B_{y}}{B_{y}}\;=\;alnB_{y}+b$$ (10)

for the two cases of the density dependence of the SPR. The Eqs (9) and (10) may be solved for B_y_ to obtain its estimate when B_y+1_ and C_y_ are available. The process of solving the equations and estimating the successive B_y_ values may be continued backwards until the year with the earliest available catch volume. Solving Eq (10) for B_y_ may be done only numerically, and it may be easily implemented in a spreadsheet. Generally, two solutions of Eqs (9) and (10) for B_y_ exist, and it is suggested to use the one that is closer to estimates of B_y+1_.

### Using differential stock-production models to estimate biomass in the pre-assessment era

The Eqs (7) and (8) define dependence of the SPR on biomass and result from the difference form of the Schaefer and Fox stock-production models, respectively. Now the differential form of the Schaefer [13] stock-production model will be used as a tool for estimating biomass in the pre-assessment era

$$\frac{dB}{dt}\;=\;HB(B_{\infty }-B)-qEB$$ (11)

where parameter H is ratio of intrinsic rate of increase to asymptotic biomass. First, the model was fitted to current assessment results from ICES, i.e., to the observed catch volumes and estimated biomass. Fletcher parameterization [16] was applied to fit the Schaefer model. In this parameterization, the maximum productivity m, rather than the parameter H, is estimated. The advantage of such parameterisation is that maximum productivity, which is parameter of main interest as equal to MSY (maximum sustainable yield) is directly estimated. Parameters m and H are related through the equation H = 4m/B_∞_^2^. For the fishing effort, the fishing mortalities averaged over fully recruited ages within the year (the so-called F_bar_) were taken. The model parameters were estimated by minimizing the sum of the squared differences between the logged “observed” values and the production model estimated values for catch volumes and biomasses. Biomass estimates provided from ICES assessment were taken as “observed” values. The relation between B_y+1_ and B_y_ in the Schaefer differential stock-production model is:

$$B_{y+1}\;=\;\frac{B_{\infty }-F_{y}/H}{1+(\frac{B_{\infty }-F_{y}/H}{B_{y}}-1)e^{-HB_{\infty }+F_{y}}}.$$ (12)

The F_y_ may be approximated by 2C_y_ /(B_y+1_+B_y_) and after obtaining the parameters of the Schaefer model Eq (12) may be solved for B_y_ numerically.

However, the above procedure of B_y_ reconstruction is quite complex and in the present application, a modification of the production model was used in the backwards reconstruction of biomass. The formulae for the production model were modified similarly to the stock numbers in Pope’s study [17] on cohort analysis compared to the classical VPA approach. Assuming that the catch takes place exactly in the middle of the year (thus, F is zero up to time t+1/2), the biomass at half of the year is:

$$B_{y+1/2}\;=\;\frac{B_{\infty }}{1+(\frac{B_{\infty }}{B_{y}}-1)e^{-HB_{\infty }/2}},$$ (13)

and by subtracting yearly catch from that biomass and again assuming zero F in the next half of the year, the biomass at the end of the year equals:

$$B_{y+1}\;=\;\frac{B_{\infty }}{1+(\frac{B_{\infty }}{B_{y+1/2}-C_{y}}-1)e^{-HB_{\infty }/2}}.$$ (14)

In backward calculations, first, B_y+1/2_ is estimated from Eq (14):

$$B_{y+1/2}\;=\;\frac{B_{\infty }}{1+(\frac{B_{\infty }}{B_{y+1}}-1)e^{HB_{\infty }/2}}+C_{y}$$ (15)

and next, B_y_ is obtained using Eq (13):

$$B_{y}\;=\;\frac{B_{\infty }}{1+(\frac{B_{\infty }}{B_{y+1/2}}-1)e^{HB_{\infty }/2}}$$ (16)

### Testing the methods

First, the procedures were tested on those stocks for which analytical assessment covers 5 to 6 decades and the tests consisted of the following steps:

- Separation of the data from the analytical assessment (here, by data, we mean catch volumes, biomass and fishing mortality estimates) into two time periods: FIRST (covering the most recent data, usually 3 decades), and SECOND (covering the earlier data).
- Fitting the SPR and Schaefer differential stock-production model to the FIRST part of the data from the analytical assessment.
- Application of the developed methods and the constant SPR method for the reconstruction of the biomass in the SECOND period.
- Comparison the reconstructed biomass with biomass estimates from the analytical assessment and concluding on how different methods have performed in such tests.

The performance of each method was measured by a root mean square relative error, RMSE, defined as

$$RMSE\;=\;(1/n\sum {{\left(\frac{B_{reconstructed}{-B}_{ICES}}{B_{ICES}}\right)}^{2})}^{1/2},$$ (17)

and mean relative error (to measure bias of the reconstructed biomass) calculated as

$$MRE\;=\;1/n\sum \frac{B_{reconstructed}{-B}_{ICES}}{B_{ICES}},$$ (18)

where n is number of years with reconstructed biomass.

Similar testing procedure was applied for stock for which biomass was to be reconstructed. The performance of the methods in the testing procedure was analyzed and biomass were reconstructed using ALL assessment data or data from the selected period (e.g., from the SECOND period if the testing procedure had shown marked differences in the results obtained from the SECOND and FIRST periods). The final estimate of the reconstructed biomass levels was the weighted average (inverse of variance weighting) of the estimates derived from the applied methods. If some of the methods performed unrealistically or very badly, they were excluded from the average.

The variance of the reconstructed biomass levels was estimated using parametric bootstrapping. In the case of the SPR based methods, the bootstrap replications of SPR were obtained by adding to the SPR used in Eq (2) or Eqs (7)–(10) a normally distributed random error with zero mean and variance σ^2^. For the constant SPR method σ is taken as standard deviation of the SPR values estimated from ICES assessment, while for the biomass dependent SPR σ is assumed as the regression standard error in Eqs (7) and (8).

For reconstructions using the differential stock-production model, the bootstrap replications of biomass were obtained by multiplying reconstructed biomass by exp(error), where error is normally distributed random variable with zero mean and variance of the Schaefer model fit to the ICES data (see S1 Supplementary Data).

### Forward calculations

For both the surplus production rate methods and the differential stock-production model method, the backward calculations for biomass reconstruction may be replaced by a forward estimation procedure. Then, the main parameter in question is the initial biomass, B_0_, i.e., the biomass level in the first year when catch volume data are available. Eqs (9), (10), (13) and (14) are then used to estimate B_y+1_ from the values of B_y_, C_y_ and the parameters. Thus, assuming a value for B_0_, we can estimate the sequence of B_y_. B_0_ may be selected such that the biomass level calculated for the first year with analytical assessment is as close to this analytical biomass as possible. This may be done by minimizing the squared difference between the modelled and already available estimate of the biomass.

### Stocks for which reconstruction was performed

The constant SPR and the other methods developed above were tested on Arctic cod and North Sea herring, for which analytical assessments cover the period from 1946 onwards (1947 for herring). Next, these methods were applied to the North-east Atlantic mackerel, as the reconstructed biomass of this stock was needed to fulfil specific tasks of the FP7 project EURO-BASIN (Contract 264 933). The analytical assessment of mackerel refers to 1972–2012, while the reconstruction was to cover the period from the early 1950s onwards. The catch volumes of mackerel for that period were taken from the ICES database. To perform the calculations, Excel macros and Visual Basic programs were developed.

ICES assessment of these stock has been age-based, however, different models were used. For Arctic cod assessment ICES applied Extended Survivors Analysis (XSA) [18]. This is one of the most often used assessment models by ICES and it assumes that catches are exact. ICES assessment of North Sea herring uses SAM model [19]. In this assessment method fishing mortality is modelled by a random walk. To assess mackerel stock ICES used Integrated Catch Analysis (ICA) model [20]. In this model fishing mortality in most recent years is assumed to be separable on year and age effect. Common feature of ICES assessments of these stocks is age basis, while the reconstruction methods used here do not apply age information.

## Results

### Arctic cod tests

**Fig 1:**
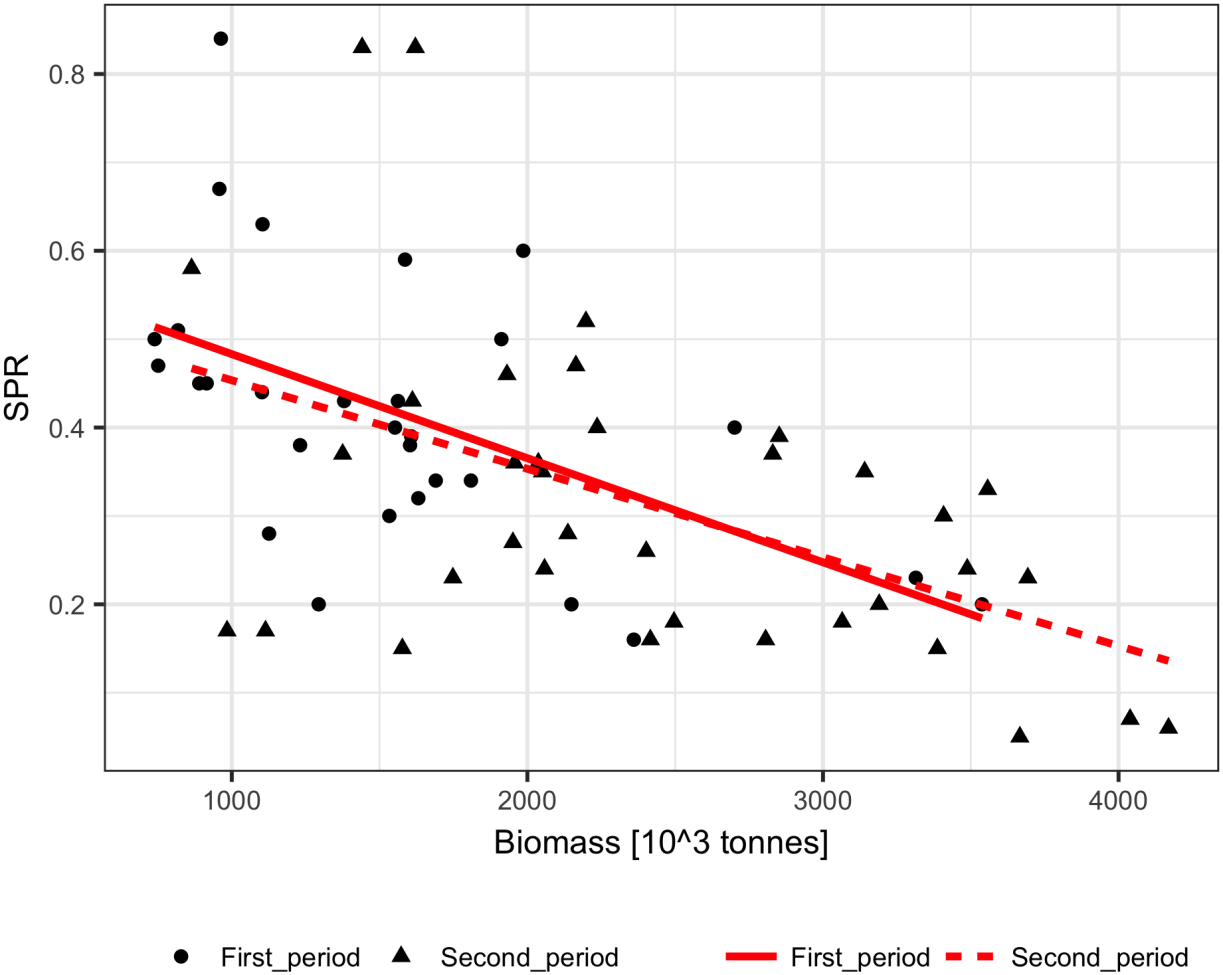
The surplus production rate, SPR, as a function of the biomass of Arctic cod (1982–2011, First_period; 1946–1981, Second_period). Red lines show linear regressions fitted to the observed SPR.

**Fig 2:**
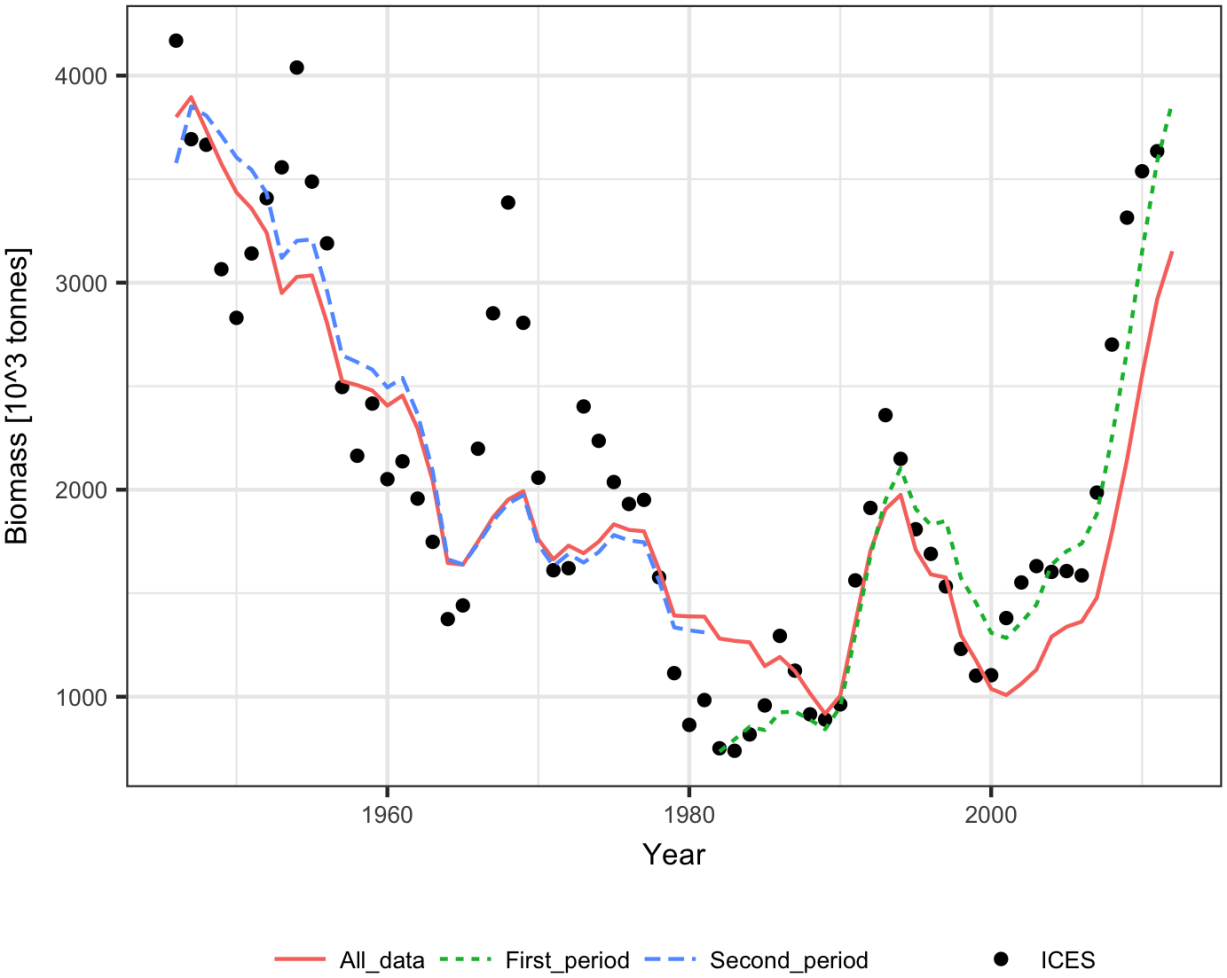
Biomass of Arctic cod fitted by the Schaefer model to the ICES analytical estimates of biomass for the First, Second, and All data years.

**Fig 3:**
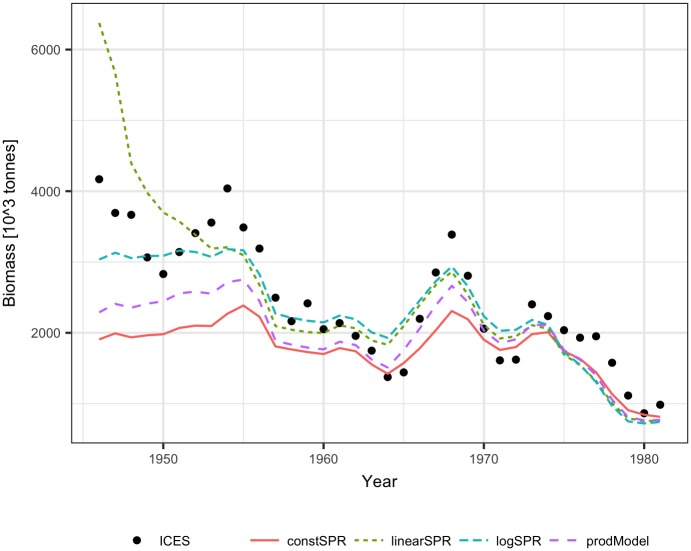
Reconstructed biomass of Arctic cod for 1945–1981 using the ICES biomass estimates for 1982–2011 and the constant SPR, linear SPR, logarithmic SPR and stock-production model methods (for comparison, ICES biomass estimates are shown).

The performance of the methods was tested using ICES assessment data from 1982–2011 (FIRST period) [21] and catch volumes from 1946–1981 (SECOND period). The dependence of the SPR on biomass was similar for the FIRST and SECOND periods, showing a significant (p<0.001) decline with biomass for both linear and logarithmic relationships (Fig 1). The biomass explained 25% to 28% of the SPR variance. The fit of the Schaefer model to the ICES assessment data explained 65%-90% of the cod biomass variance and produced quite similar model parameters estimate (B_inf_ and m) for the FIRST, SECOND, and ALL data (Table 1, Fig 2). The reconstruction results and the ICES assessment are presented in Fig 3. Up to the mid-1950s, all the methods reconstructed biomass relatively well, and the largest differences in method performance can be seen for the earliest 10 years. The logarithmic SPR method performed best (RMSE = 0.19 and MRE = -0.04), while the constant SPR method markedly underestimated cod biomass (RMSE = 0.27 and MRE = -0.22, Table 2). The reconstructions of biomass by the linear SPR and stock-production methods differed from the ICES biomass moderately, showing RMSE values of 0.22–0.23 and MRE from 0.01 to -0.16.

**Table 1:** The carrying capacity, B_inf_, (10^3^ t.) and maximum productivity, m, (10^3^ t.) for cod, herring, and mackerel estimated by the Schaefer model for the First, Second, and All data years. The standard errors (SE) of the model fits are also provided.

Stock		First	Second	All
Arctic cod	m	879	800	762
	B_inf_	6155	5517	4979
	SE	0.18	0.22	0.37
NS herring	m	618	604	555
	B_inf_	2346	3626	3131
	SE	0.22	0.30	0.42
NEA mackerel	m	840	838	831
	B_inf_	4532	4476	4617
	SE	0.15	0.22	0.29

**Table 2:** Root mean squared relative error (RMSE) and mean relative error (MRE) of reconstructed stock biomass by reconstruction method tested for Arctic cod and North Sea herring. NA = not available; model failed to reproduce biomass before 1965.

	Arctic cod	Arctic cod	North Sea herring (till 1965)	North Sea herring (till 1965)	North Sea herring	North Sea herring
method	RMSE	MRE	RMSE	MRE	RMSE	MRE
constSPR	0.27	-0.22	0.87	0.73	0.65	0.21
linearSPR	0.23	0.01	0.29	-0.02	NA	NA
logSPR	0.19	-0.04	0.4	-0.26	0.98	0.46
prodModel	0.22	-0.16	0.45	0.14	0.66	0.44

**Fig 4:**
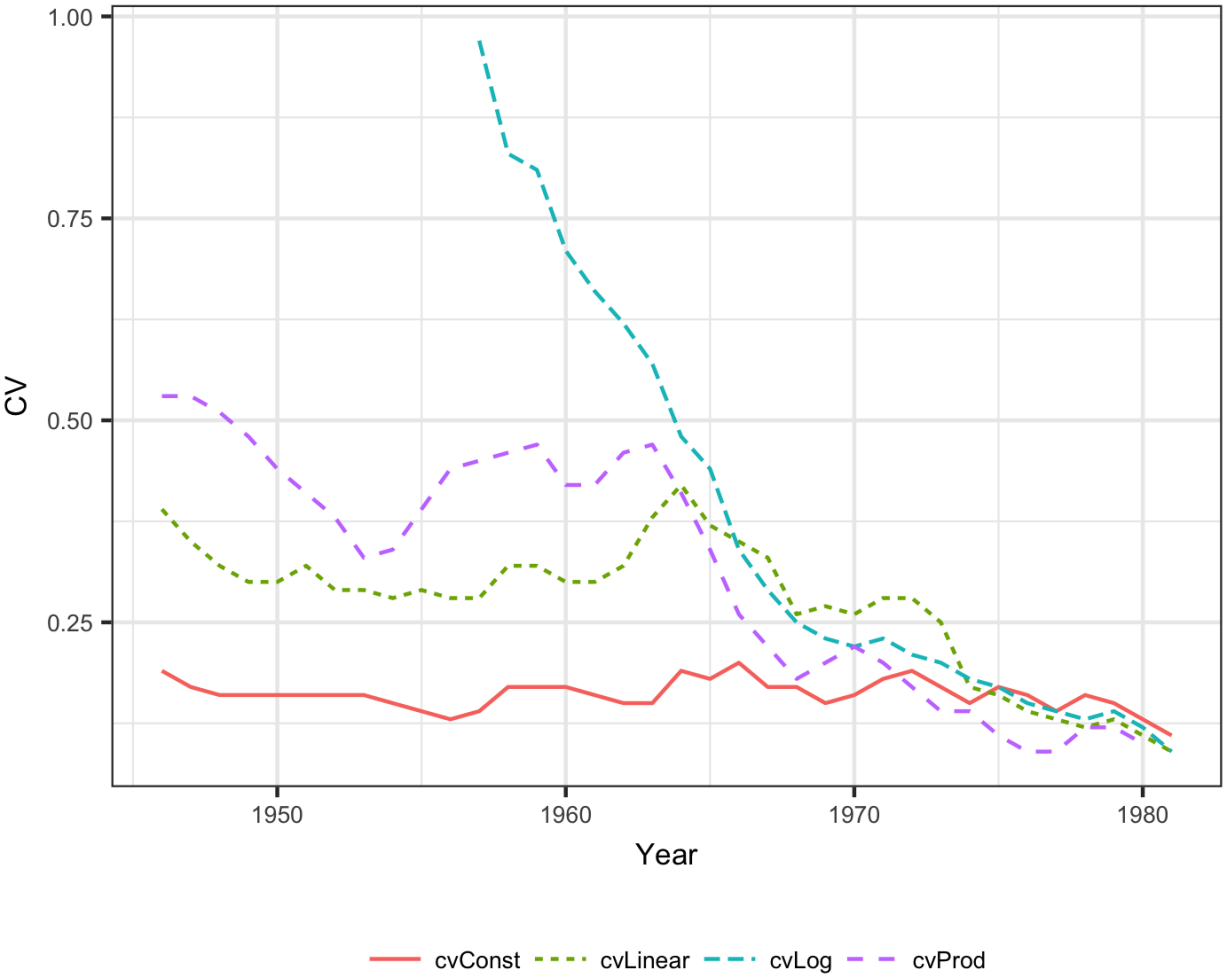
The CV of the reconstructed biomass of Arctic cod obtained from the constant SPR, linear SPR, logarithmic SPR and stock-production model methods.

**Fig 5:**
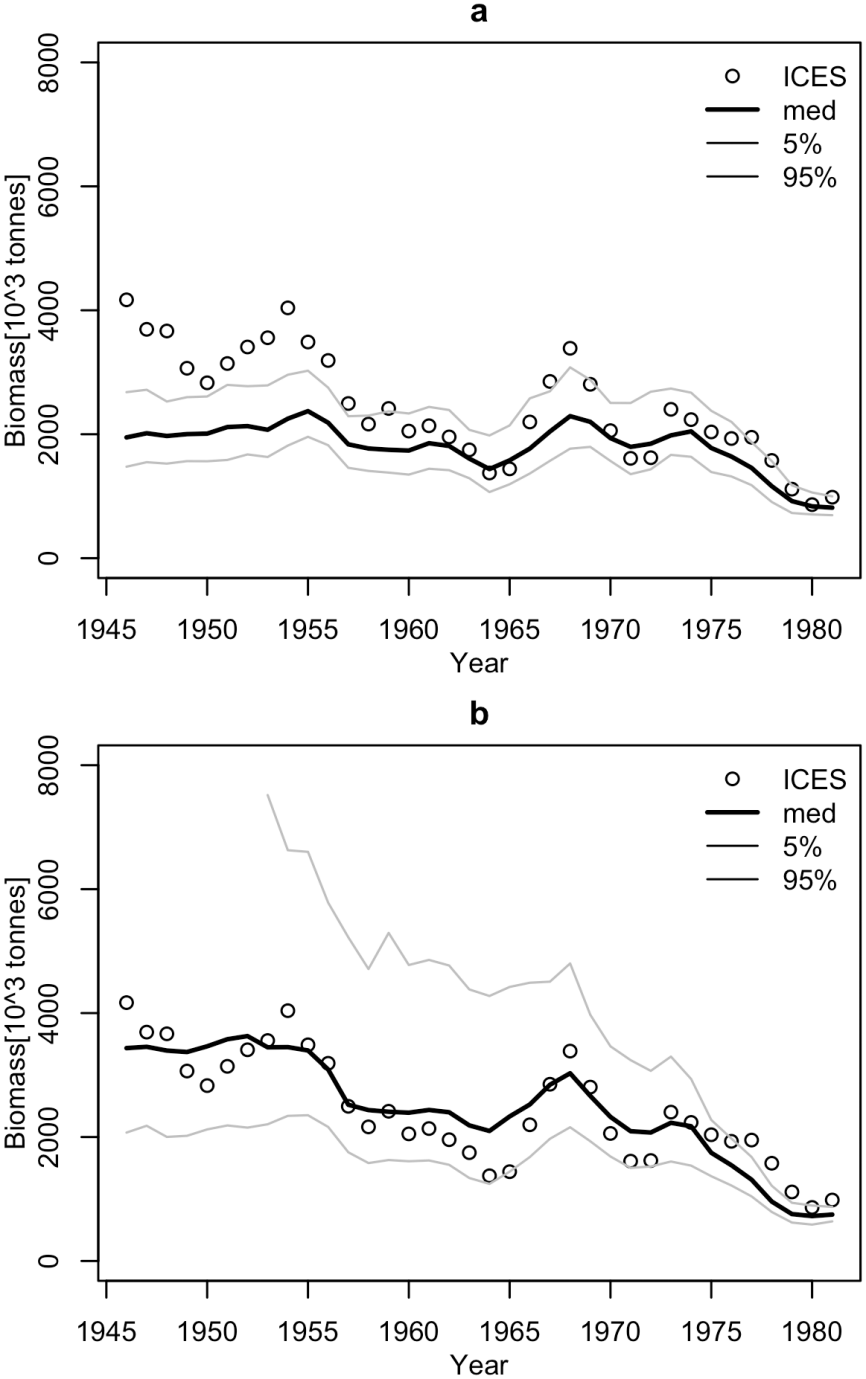
The median and percentiles of Arctic cod biomass distribution, reconstructed using the constant SPR (a) and logarithmic SPR (b) methods.

The lowest CV of reconstructed biomass was obtained for the constant SPR method, while the logarithmic SPR method provided the highest CV of reconstructed biomass, reaching 3.5 in the earliest years (Fig 4). However, the constant SPR method led to biased estimates of reconstructed biomass, and the 90% confidence intervals estimated by bootstrapping did not cover the true biomass levels (Fig 5a). The biomass CVs in other methods were much higher, but the true biomass was constrained within 90% confidence intervals of reconstructed values, as shown (Fig 5b) for the logarithmic SPR method.

### North Sea herring tests

**Fig 6:**
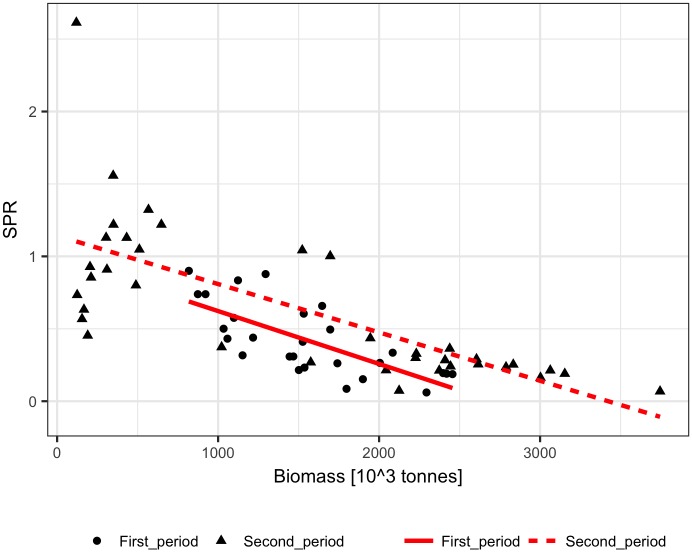
The surplus production rate, SPR, as a function of the biomass of North Sea herring (1982–2011, First_period; 1947–1981, Second_period). Red lines show linear regressions fitted to the observed SPR.

**Fig 7:**
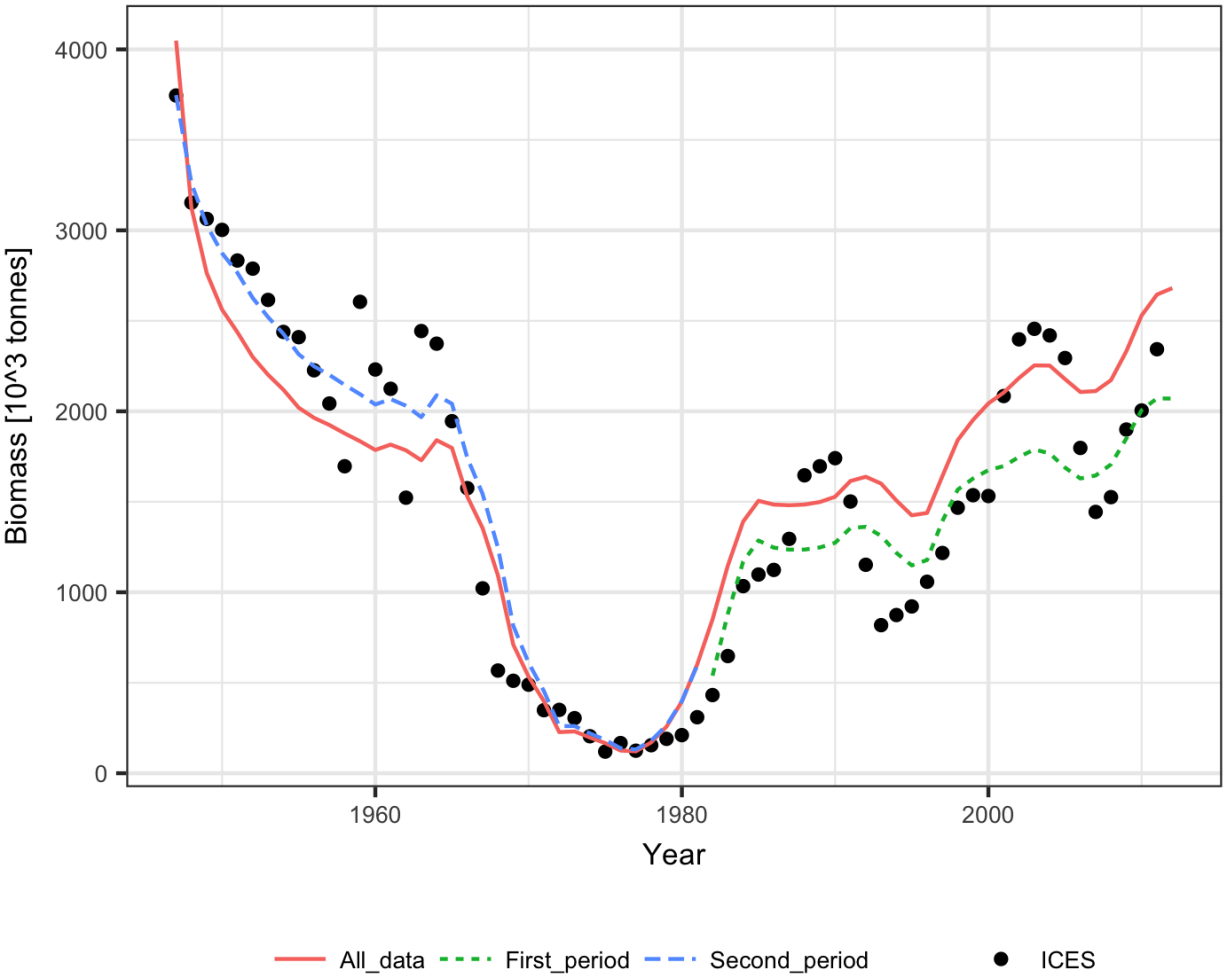
Biomass of North Sea herring fitted by the Schaefer model to the ICES analytical estimates of biomass for the First, Second, and All data years.

**Fig 8:**
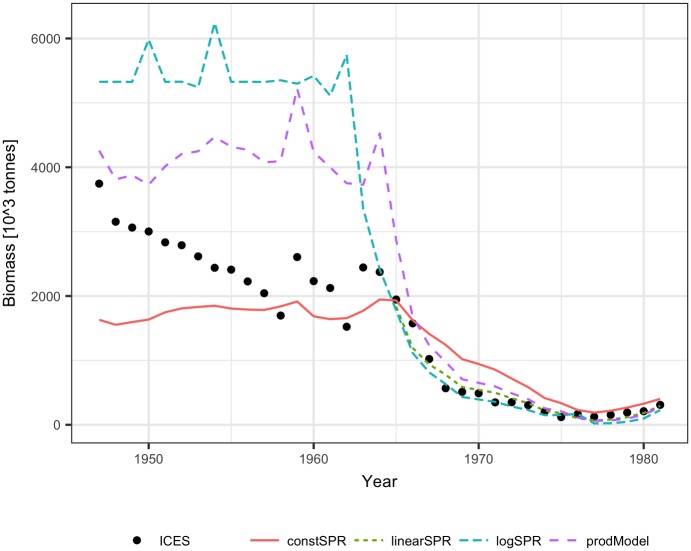
Reconstructed biomass of North Sea herring for 1947–1981 using the ICES biomass estimates for 1982–2011 and the constant SPR, linear SPR, logarithmic SPR and stock-production model methods (for comparison, ICES biomass estimates are shown).

The performance of the methods was tested using ICES assessment data from 1982–2011 (FIRST period) [22] and catch volumes from 1947–1981 (SECOND period). The SPR significantly declined with biomass in the FIRST and SECOND periods for both linear and logarithmic relationships (p<0.001), but the slopes of the SPR-biomass relationship were somewhat different (Fig 6). The biomass explained approximately 50% to 60% of the SPR. The fit of the Schaefer model to the ICES assessment data explained 70% to 90% of the herring biomass variance and produced similar estimates of maximum productivity m and somewhat different estimates of B_inf_ for the FIRST, SECOND, and ALL data (Table 1, Fig 7). The biomass reconstruction was based on the ALL assessment data points. Up to the mid-1960s, the density dependent SPR methods and the stock-production model reconstructed biomass quite well (RMSE in range 0.29–0.45, RME from -0.26 to 0.14), while reconstruction by the constant SPR method performed worse (RMSE of 0.87 and MRE of 0.73) (Table 2, Fig 8). For earlier years, however, the biomass reconstruction was very imprecise, and the constant SPR method performed somewhat better than the other approaches. The RMSE and MRE for the whole period were lowest for the constant SPR method (0.65 and 0.21, respectively), while for the other methods RMSE ranged from 0.66 to 0.98 and MRE was at 0.44–0.46.

### Mackerel tests

**Fig 9:**
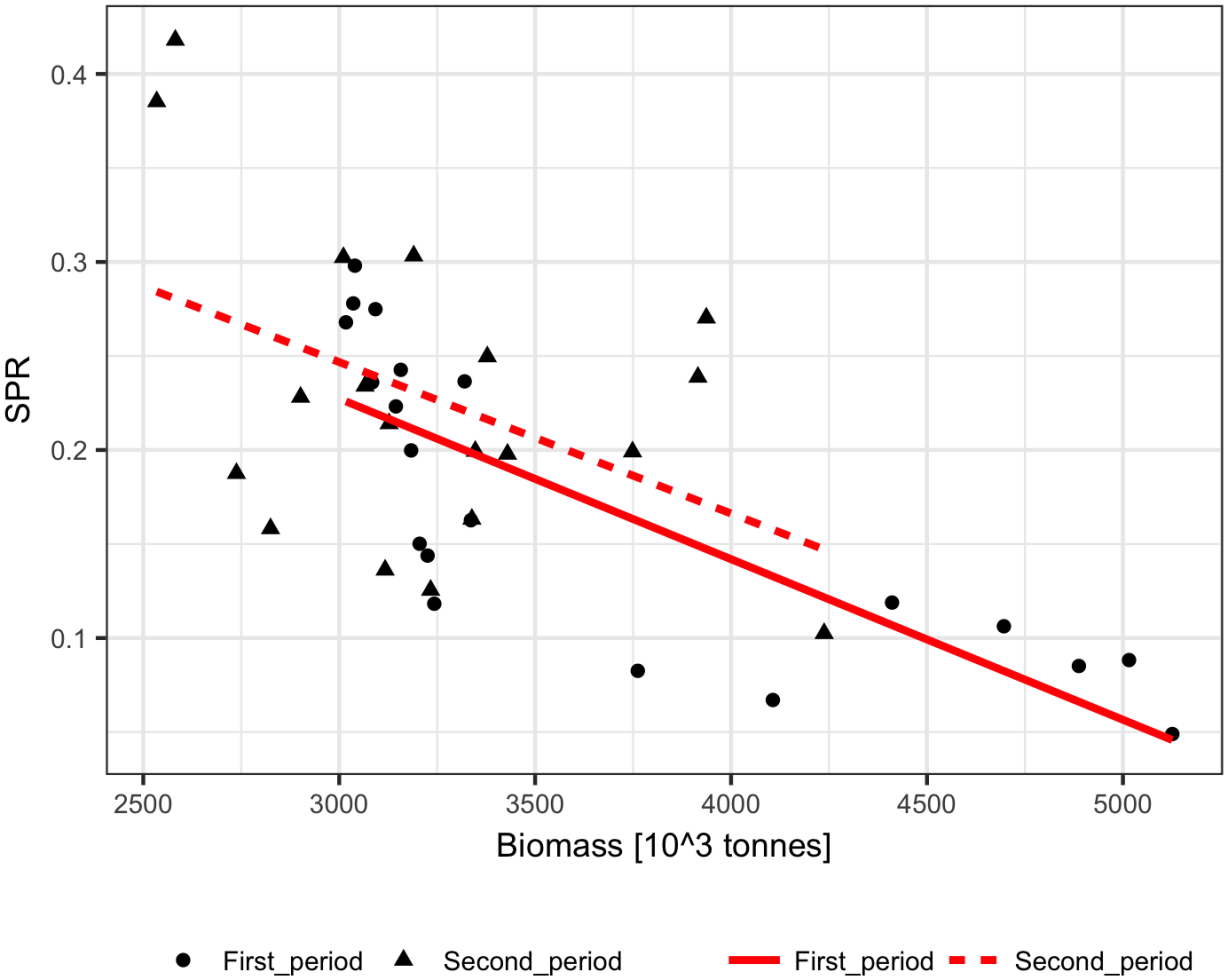
The surplus production rate, SPR, as a function of the biomass for mackerel (1992–2011, First_period; 1972–1991, Second_period). Red lines show linear regressions fitted to the observed SPR.

**Fig 10:**
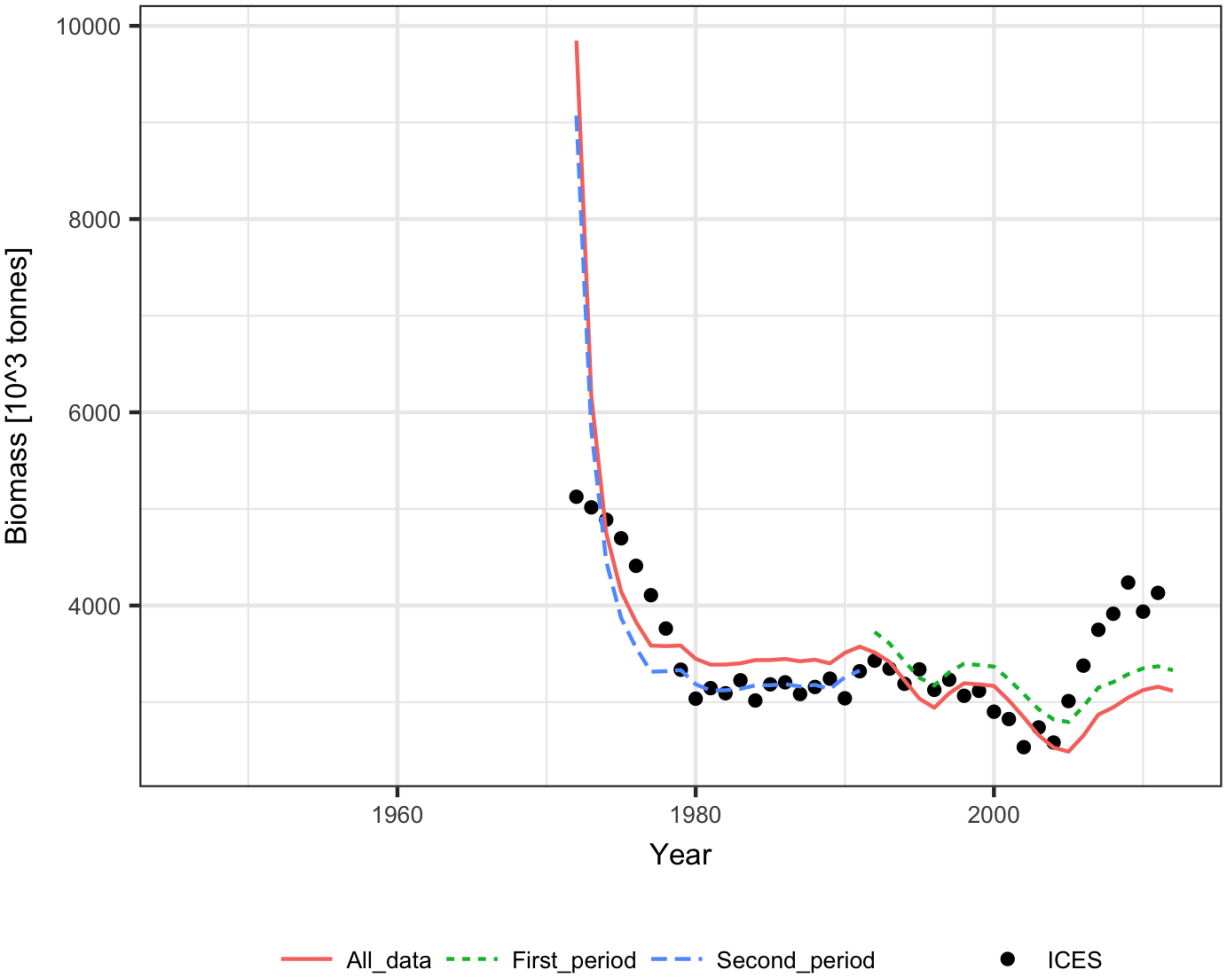
Biomass of mackerel fitted by the Schaefer model to the ICES analytical estimates of biomass for the First, Second, and All data years.

The ICES assessment of mackerel covered the years 1972–2011 [23]. The available catch volume data extended backwards to 1950, and the biomass was reconstructed up to 1950. To test the reconstruction methods for that stock, the assessment data were separated into two periods, a FIRST period covering the years 1992–2011 and the SECOND period covering the years 1972–1991. The dependence of the SPR on stock size was quite similar for the FIRST and SECOND data, and the SPR significantly declined with biomass for both linear and logarithmic relationships (p<0.03) (Fig 9). The logarithm of the biomass explained slightly more variance of the SPR than the biomass itself, and a much better fit of the SPR was obtained for the SECOND data than for the FIRST data (66% and 23% of the explained variance, respectively). The stock-production model fitted the ICES assessment data for the FIRST, SECOND, and ALL data years relatively well; only the biomass estimate for the initial year (1972) deviated much from the ICES analytical biomass, and the estimates for the next years were close to the analytical values (Fig 10). The estimated parameter values were very similar for the considered periods (Table 1).

The backward estimation with density dependent SPR methods and the stock-production model method applied for the biomass reconstruction worked only for a few years, producing strongly increasing estimates, before the methods failed. In the case of the density dependent SPR, Eqs (9) and (10) did not have a solution, while the production-model method produced unrealistic values. Therefore, forward estimation procedures were used for testing the performance of both methods and for biomass reconstruction.

**Fig 11:**
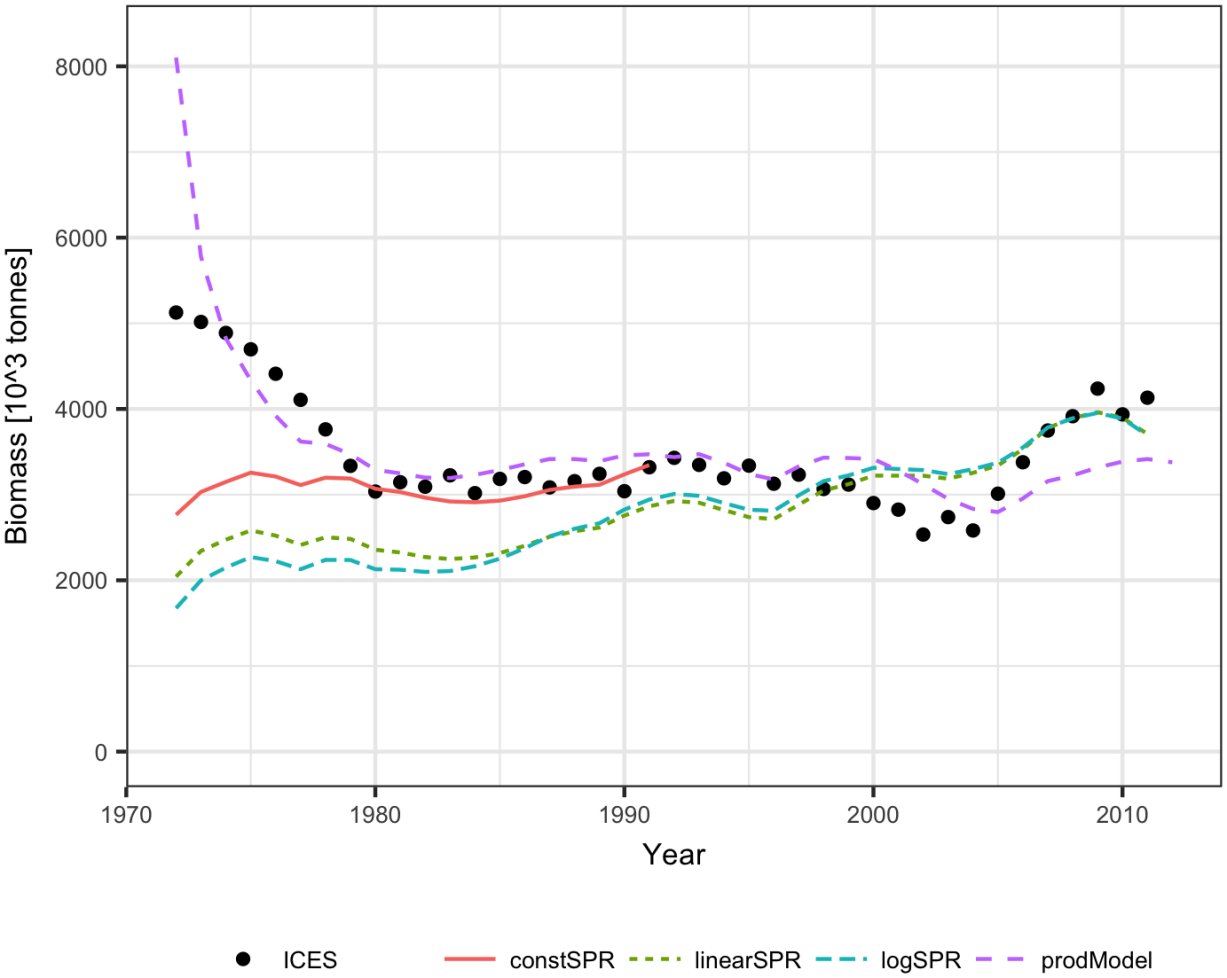
Testing the methods for mackerel: Reconstructed biomass for 1972–1991 using the ICES biomass estimates for 1992–2011 and the constant SPR, linear SPR, logarithmic SPR and stock-production model methods (for comparison, ICES biomass estimates are shown).

When using the FIRST assessment data and the density dependent SPR, reconstructed biomass levels were lower than the analytical biomass, but they showed a similar trend from 1978 onwards (Fig 11). For earlier years, the reconstructed biomass declined, while analytical biomass went up. The constant SPR method produced biomass estimates closer to the analytical biomass than the estimates from the density dependent SPR, but these estimates also declined for years before 1978. The reconstruction of biomass using the FIRST assessment data and the stock-production model performed much better than the SPR methods (only the estimate for 1972 deviated largely form the analytical biomass) (Fig 11).

**Fig 12:**
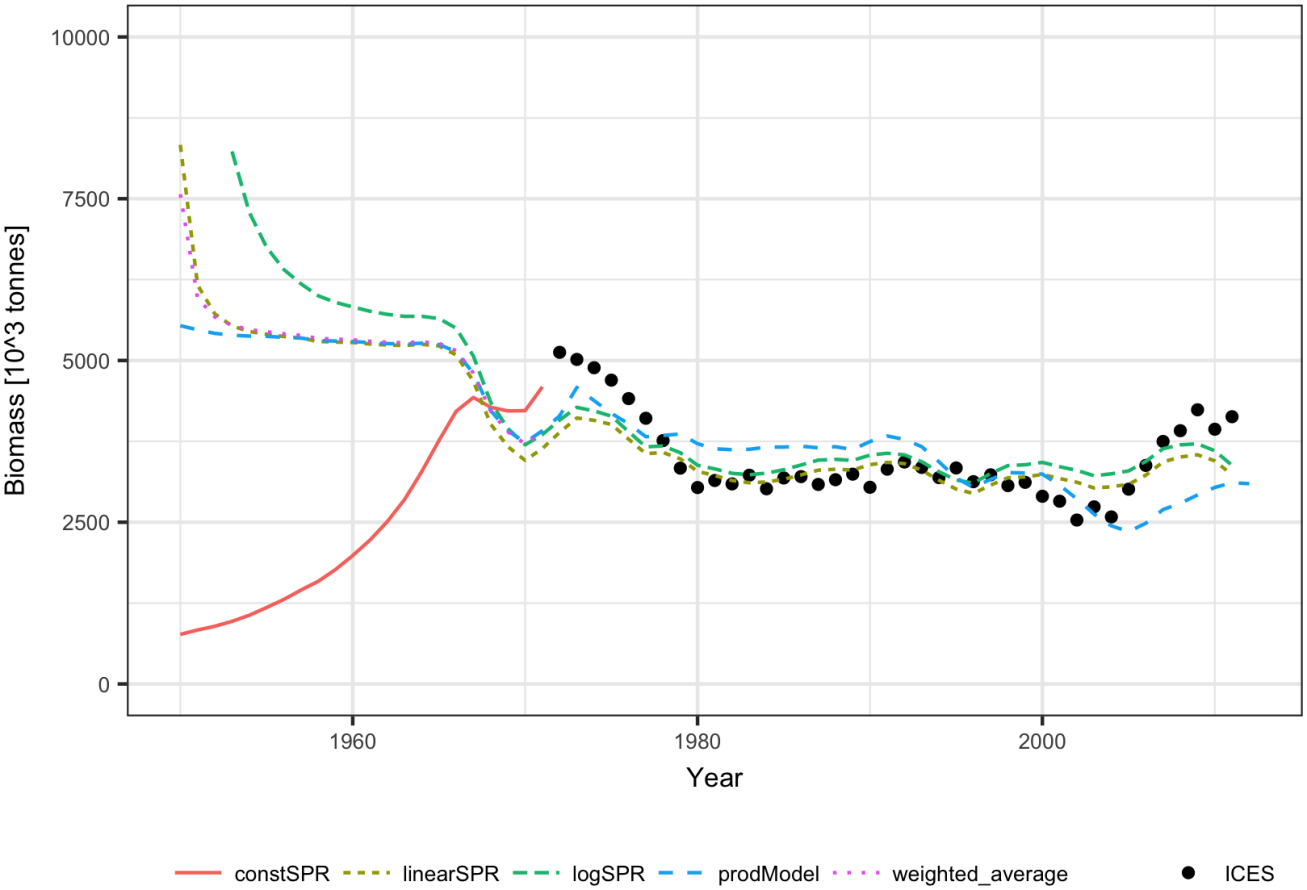
Reconstructed biomass of mackerel for 1950–1971 using the ICES biomass estimates for 1982–2011 and the constant SPR, linear SPR, logarithmic SPR and stock-production model methods.

The SPR based models and the Schaefer model fitted to the ALL data points were used as a basis for the reconstruction of biomass in the 1950 to 1971 period (Fig 12). The density dependent SPR methods showed a backward increasing trend of biomass estimate, while the constant SPR method showed the backward estimated biomass declining to zero. The stock-production model method produced reconstructed biomass levels similar to those of the density dependent methods for all years except the earliest. The results of the constant SPR method were considered unrealistic; therefore, the weighted average of estimates from the density dependent SPR methods and the stock-production model method were used as a final estimate.

## Discussion

Methods were developed for the reconstruction of stock biomass in years when only catch volume was available. The methods may be applied when the SPR is density dependent, which makes them applicable in wider conditions than the constant SPR method of Eero and McKenzie [12]. Tests of the reconstruction methods on stocks with a long period of analytically estimated biomass showed quite satisfactory performance for the developed approaches. The constant SPR method may fail if catch volumes during the reconstructed period are low (mackerel example).

The stock density dependent SPR methods are, to some extent, similar to the production model approach used for biomass reconstruction because the relationship between the SPR and biomass was developed based on the discrete version of the stock-production models. However, the analysis performed with the production model used the differential form of the Schaefer model. Consequently, results from both approaches were different, and the North Sea herring example even showed that one approach may perform relatively well (the production model approach), while the other (the linear SPR method) may fail.

Both backward calculations and forward procedures were developed and used to perform biomass reconstruction. The disadvantage of the forward procedure compared with backward estimations is the need to estimate an additional parameter, B_0_, the biomass in the first year. In addition, the data sets used in the present paper have shown that estimation results may be very sensitive to the choice of B_0_ because very small deviations from the “exact” B_0_ value may lead to very different series of estimated biomass. However, backward calculations may not have a solution or can lead to unrealistic results after a few years of back-calculations, as has been shown in the mackerel example. In this paper, the forward estimation procedure fitted B_0_ by minimizing the difference between the reconstructed biomass and the analytical biomass in the first year for which the latter was available. This may be replaced by minimization of the differences between longer time series of biomass levels provided by an analytical assessment and the biomass levels obtained in forward estimations. Such an approach could be justified, e.g., when the analytical biomass estimate for the first year has greater variance than the estimates of biomass in the next years.

In the mackerel test case, application of the constant SPR method provided biomass estimates declining to zero. This result is easily explained when the average SPR is calculated for a period in which it is relatively high (which is often the case for the “assessment era,” usually including years of rather high exploitation) while catch volumes in the reconstructed period are low. In this case, the denominator in Eq (2) is markedly larger than 1 (1.2 for mackerel), while the numerator is close to B. In such a case, Eq (2) may be approximated by *B*_*y-n*_ = *B*_*y*_*/(1+SPR)*^*n*^ (whereby the biomass in year *y-n* depends on the biomass in year *y* and the SPR only), which tends to zero for *n* increasing to infinity.

The reconstruction of stock biomass is not a simple task, and as shown by the examples provided, each of the tested methods may fail for a specific stock and a specific period. The approach for biomass reconstruction should be stock and available-data specific, and data other than only catch volume and analytical assessment data could be helpful and used for biomass reconstruction. For example, Eero [24] extended estimates of Baltic sprat biomass to the 1930s. First, she extended the analytical assessment for several years, and next, she derived a relationship between the biomass from the analytical stock assessment and the stock size indices from an eggs survey. That relationship was applied to extrapolate the sprat stock size in selected years from 1930 to the 1950s, for which egg survey indices were available. Cardinale et al. [25] used an otter trawl survey, which started in 1906, to estimate the relative abundance and spatial distribution of haddock and pollack in the Kattegat and Skagerrak areas during the previous century. General Additive Models (GAMs) were applied to standardize survey data and to evaluate the spatial distribution of biomass.

Stock-production models have already been used for reconstructing biomass. Rose [26] used a simple difference form of the Schaefer model, assuming that Atlantic cod was at its carrying capacity in 1505, and reconstructed cod biomass for approximately 500 years. The reconstruction of whale biomass was also mostly based on some variant of logistic stock-production models, assuming that stock size was at carrying capacity at the beginning of the exploitation and reconstructing biomass in the forward mode using known catch volumes [27]. Such approaches are similar to the forward procedure developed in the present paper, with the exception that here, the initial biomass was estimated. Witting [28, 29] used a few age and sex structured dynamics models to analyse delayed density dependent feedbacks in baleen whales. Applying these models, he estimated the stock size of some North Atlantic right whale and eastern Pacific grey whale from the 17^th^ century onwards. However, the goal of the above simulations was not essentially biomass reconstruction but rather an investigation of feedback mechanisms, and the overall conclusions were not sensitive to the assumption of an initial stock size at carrying capacity. Kimura and Tagart [30] developed the stock reduction analysis (SRA), which could be considered as an alternative method of biomass reconstruction using forward calculations. The strength of SRA increases when it is coupled with Deriso delay-difference model [31, 32]. Recently developed a Bayesian state space model [33] is an extension of SRA and could be also applied as an alternative method of biomass reconstruction using forward calculations. These approaches apply difference version of the models while our approach is based on differential solution of production models.

It is difficult to evaluate the current state of marine ecosystems or to make future projections without knowing about the history, magnitude and drivers of past changes [34–37]. As stated in Lotze and Worm [34], until recently, marine ecology, conservation and management focused largely on the last 20–50 years of scientific monitoring data but rarely provided historical reference points that reach back to the beginning of exploitation. The developed methodologies for the reconstruction of stocks biomass where only the catch data are available deliver tools to full fill that gap and reach back in time when only fisheries statistics are available. Presented methodology is our contribution in to understanding of ocean past effort, allow to put current trends at fish stocks in to long term non shifted-baseline perspective [34,38] and inform management and conservation efforts.

## Supporting information

S1 Supplementary Data Data set underlying the input data and findings in study in the manuscript “Extension of biomass estimates to pre-assessment periods using density dependent surplus production approach” by Jan Horbowy and Maciej T. Tomczak. (XLSX)
